# Altruism in Forest Chimpanzees: The Case of Adoption

**DOI:** 10.1371/journal.pone.0008901

**Published:** 2010-01-27

**Authors:** Christophe Boesch, Camille Bolé, Nadin Eckhardt, Hedwige Boesch

**Affiliations:** 1 Department of Primatology, Max Planck Institute for Evolutionary Anthropology, Leipzig, Germany; 2 Centre Suisse de Recherche Scientifique, Abidjan, Côte d'Ivoire; Yale University, United States of America

## Abstract

In recent years, extended altruism towards unrelated group members has been proposed to be a unique characteristic of human societies. Support for this proposal seemingly came from experimental studies on captive chimpanzees that showed that individuals were limited in the ways they shared or cooperated with others. This dichotomy between humans and chimpanzees was proposed to indicate an important difference between the two species, and one study concluded that “chimpanzees are indifferent to the welfare of unrelated group members”. In strong contrast with these captive studies, consistent observations of potentially altruistic behaviors in different populations of wild chimpanzees have been reported in such different domains as food sharing, regular use of coalitions, cooperative hunting and border patrolling. This begs the question of what socio-ecological factors favor the evolution of altruism. Here we report 18 cases of adoption, a highly costly behavior, of orphaned youngsters by group members in Taï forest chimpanzees. Half of the adoptions were done by males and remarkably only one of these proved to be the father. Such adoptions by adults can last for years and thus imply extensive care towards the orphans. These observations reveal that, under the appropriate socio-ecologic conditions, chimpanzees do care for the welfare of other unrelated group members and that altruism is more extensive in wild populations than was suggested by captive studies.

## Introduction

In recent years, extended altruism towards unrelated group members has been proposed to be a unique characteristic of human societies [1]–[8]. Evolutionary theory predicts that altruistic interactions, which are costly to the actor and beneficial to the recipient, will be limited to kin or reciprocating partners [1]–[2]. In contrast to such predictions, economists adopting a rational maximizing approach were struck by the fact that experiments done in different human societies did not support such a model. Rather, humans were always willing to share or cooperate with others more than expected [3], [5]–[8]. This resulted in an effort to identify the mechanisms that would lead to such observations and, in the end, it was proposed that both punishment by one's peers and reputation improvement will promote altruism towards unrelated group members in humans [3]–[5]. In a complementary approach, experimental studies done with captive chimpanzees showed limits in the way individuals were able to share or cooperate with others, especially when it came to food [9]–[15]. This dichotomy between humans and chimpanzees was proposed to indicate an important difference between the two species, and one study proposed that “chimpanzees are indifferent to the welfare of unrelated group members” [9]. This difference was suggested to result from chimpanzees' inability to think about others' minds and therefore understand that others might need or could profit from help [13]–[15].

In strong contrast to these studies with captive chimpanzees, consistent observations of potentially altruistic behaviors in wild chimpanzees have been reported from different populations in such different domains as food sharing, regular use of coalitions, and cooperative hunting and border patrolling [16]–[20]. The striking differences between captive and wild populations beg the question of what socio-ecological factors favor the evolution of altruism within one species. From an evolutionary standpoint, Hamilton's rule proposes that altruism should be favored under two conditions: 1) when the cost of the altruistic act is compensated by the genetic relatedness between the giver and the receiver, and 2) when the cost of the altruistic act will be compensated at a later time through reciprocation [1]–[2]. If altruistic acts have often been observed to occur between closely related animals [21]–[22], it is the occurrence of such acts between unrelated individuals that was proposed to be uniquely human [2]–[5]. Concerning the first condition, it is important to note that in natural social groups of chimpanzees, the impact of kinship has been shown to be rather limited as the vast majority of dyads are unrelated [23]–[24]. The second condition is more difficult to evaluate as the reciprocation can either occur over a relatively extended period of time if the species considered has the required cognitive capability, or it can occur through exchange with another commodity. For example, Taï male chimpanzees have been shown to exchange meat for mating opportunities with females on a long term basis [25]. Similarly, in a comparison of 15 different human societies, the level of altruistic sharing varied with the level of market integration and group size [6]–[8]. Following Hamilton's rule, we should expect more altruistic behavior in populations of individuals as the benefit becomes relatively larger than the cost. Thus, the proposed absence of altruistic food sharing in captive animals might be expected due to the well-fed state of all individuals under such conditions [5]–[8]. In natural conditions, we might expect many situations in which an altruistic act would increase the survival of group members, like in the cases of adoption, and defense against predators or aggressive outsiders [10], [26].

To elucidate some of the specific conditions that elicit altruism towards unrelated group members in chimpanzee social groups, we report here about regular adoption, a highly costly behavior, of young orphans by group members in forest chimpanzees in the Taï National Park, Côte d'Ivoire. Some adoptions of orphans by unrelated adult males lasted for years. Extensive parental care towards unrelated group members in chimpanzees reveals some of the socio-ecological conditions under which the evolution of altruism could be expected in social groups.

## Results and Discussion

We operationally defined adoption as any relationship between an adult and orphan infant or juvenile in which the adult shows species-specific maternal behavior towards the orphan for at least a two month period. Following a standard definition of adoption [27]–[29], we required that the adult be permanently associated with the orphan, as well as, at the very least, wait during travel for, provide protection in conflicts to, and share food with the orphan. These behaviors are altruistic in the sense that they are costly for the adopting individuals and do not bring any visible benefit to them, while being beneficial to the orphans. Since juvenile chimpanzees remain associated with their mothers for over ¾ of their time and become clearly independent of their mothers only when they have reached adolescence, we included this period in our study. It is important to underline that adult chimpanzees at Taï do not wait for juveniles or infants, or react to their whimpering at being left behind until they are their mothers. In chimpanzees, orphans suffer tremendous costs in terms of reduced survivorship (orphans less than 5 years of age normally do not survive [16], [30]) or retardation in physical development (up to 6 years delay [30]). However, if adopted, such orphans may present almost normal physical development [16].

**Figure 1:**
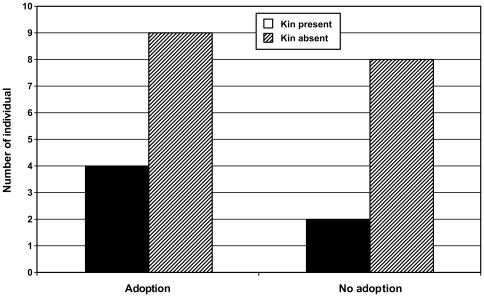
The presence of a close kin does not increase the likelihood of adoption in Taï chimpanzees (Fisher exact test: p = 0.463).

**Figure 2:**
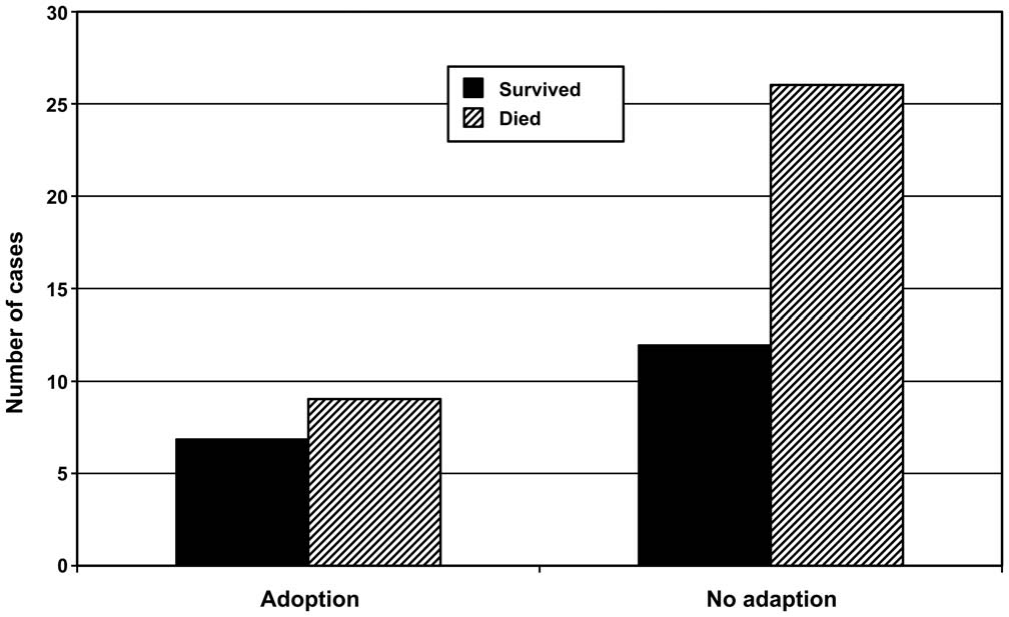
Orphans adopted do not present higher likelihood of surviving for 2 years the death of their mother than non-adopted orphans in Taï chimpanzees (X2 = 0.37, df = 1, p = 0.54).

In 3 communities of Taï chimpanzees that have been studied for 27 years, we observed 36 cases of individuals being orphaned and surviving this traumatic event for over 2 months. In 18 of these cases, an adoption was observed to occur (Table 1). In addition, during that same time interval, 22 small unweaned infants (average age of 1.85 years) lost their mothers and disappeared with them, while a handful died within a couple weeks, before an adoption was possible. Eight adoptions were performed by females, including 1 by an older sibling and 3 by possible unrelated ‘friends’ of the dead mother (Table 2). However, in general, the presence of living close relatives (e.g., full or half siblings) did not increase the likelihood of adoption in our sample (see Figure 1). Two adoption cases by adult females who breast fed unrelated young infants (less than 20 months of age) for many years are particularly noteworthy (Nabu and Totem, Table 2). These two examples are illustrative of the huge potential benefit adoption has on unweaned orphans, however, in our sample, adoption did not increase the likelihood of the orphans surviving two years following the death of their mother (see Figure 2). An equal number of male (N = 10) and female (N = 8) orphans were adopted. Orphans adopted by male rather than female adults did not differ significantly in either sex or age (Table 2).

**Table 1:** Number of orphans and adoptions seen in the 27 years of observation of three study groups of the Taï chimpanzee project.

	North Group		South Group		East Group		Total
Orphans	Male	Female	Male	Female	Male	Female	
Adopted	6	7	1	0	3	1	18
Not adopted	1	4	6	1	4	1	17

**Table 2:** Successful adoptions of orphaned infants in three communities of Taï chimpanzees.

Name of infant	Sex of infant	Age when Mother died	Name of mother	Time until adoption	Duration of adoption	Foster parent		
						Name	Sex	Relation
**North Group**								
Ali	♂	5.2 y	Awa	1 m	>5.5 y	Brutus	A♂	uk
Belle	♀	6.1 y	Biche	1.5 y	4.3 y	Pokou	A♀	uk
Bibi	♀	1.5 y	Biche	quick	5 m d1	Belle	J♀	Sister
Bonnie	♀	∼5 y	uk	*	1.1 yd2	Clyde	Ad♂	Brother
Bonnie	♀	6.5 y	Clyde	1 m	3 m^i^	Ulysse	A♂	Brother
Bonnie	♀	6.5 y	Ulysse	1.5 y	5 y	Xérès	A♀	uk
Brando	♂	4.7 y	Marlène	6.1 m	1.4 y^i^	Ulysse	A♂	NR
Chouchou	♀	6.7 y	Chanel	2 weeks	>3.4 y	Loukoum	A♀	uk
Gérald	♂	7 y	Ella	quick	>1.4 yd^1,2^	Fitz	A♂	Brother
Molière	♂	6.6 y	Momo	5 m		Kiri	A♀	Friend
Nabu	♀	10days	Nana	quick	2.5 yd1	Malibu	A♀	Friend
Sartre	♂	9.5 y	Salomé	quick	>1 yd1	Ondine	A♀	Friend
Tarzan	♂	4.9 y	Tosca	2 weeks	1.1 yd1	Brutus	A♂	uk
**East Group**								
Yayo	♂	2 y	uk	quick	4 md1	Fredy**	A♂	uk
Carim	♂	2 y	Candy	quick	4 md1	Fredy	A♂	Father
Gia	♀	2.5 y	uk	10 m	17 md2	Porthos	A♂	NR
Victor	♂	2.5 y	Vanessa	quick	7 md1	Fredy	A♂	NR
**South Group**								
Totem	♂	<2 y	uk	?	>4 y	Tita	A♀	UR

quick  =  adoption occurred within days after the death of the mother.

* =  Bonnie was first identified when she had already been adopted by her suspected brother, Clyde.

** =  Yayo has been observed to be carried by 4 other adult males of the East Group during the 4 months of his adoption, but Fredy was the main adopter.

d1 =  adoption interrupted by the death of the orphan.

d2 =  adoption interrupted by the death of adopter.

i =  adoption actively interrupted by adopter.

Relation: Sister/Brother =  older sibling of the orphan, Friend =  A♀ was a friend of the deceased mother, NR =  confirmed as not related following genetic testing, uk = unknown.

Of special interest are the adoptions by males. These cases include 3 older siblings and 6 adults performing 10 adoptions; 1 of the adoptive adult males proved to be the father of the orphan, while 3 were unrelated to the orphan, and 2 were of an unknown relationship to the orphan (Table 2). Male chimpanzees are considered to be adults when they are 15 years old. At this age, they become very social with other males of the group, spend a lot of time grooming one another, and compete aggressively for access to females. They are also the main hunters in the group and actively defend the territory from intruders [16], [30]. Male chimpanzees, like males of polygynous human societies, have not been observed to develop long-term bonds with specific females, nor to invest much in their own offspring [16], [20], [30]. Male chimpanzees at Taï have not been observed to show obvious paternal behavior, except in terms of playing more often and, to a lesser extent, grooming more with their own offspring than with other youngsters of the same age [31]. We were able to test for genetic relatedness in 4 out of the 6 cases of orphans adopted by adult males, and in 3 of them, the males were not related to the youngsters (exclusion at 2 to 4 loci were found) [32].

As can been seen in Table 3, adoption of orphans by adult males represented an important investment in the youngsters, as, minimally, males were seen to share food with them as well as wait for them and support them during social conflicts (this is the operational definition of adoption we used). The two infants adopted by the alpha male of the North Group, Brutus, matured after adoption without showing any of the physical retardation so typically seen in orphans. Therefore, they benefited dramatically from this investment. Five years into the adoption, Ali was becoming a healthy adolescent male when he died during an Ebola outbreak. Another cost of adopting orphans for males is that rivals of the adopter may use the orphan to harass him, and Ulysse, a middle-ranking male, seemed unable to cope with this and was seen to actively interrupt both the adoptions of his presumed younger sister, Bonnie, and of the unrelated male, Brando, following the increasing harassment exerted against the orphans by his rival males (Table 2).

**Table 3:** Paternal-like behavior observed during an adoption by adult males (with the maternal investment as reference).

Name of	Share	Share	Carry	Wait for	Support	Search for
the pair	Night nest	Food	Dorsally	Infant	Infant	Infant
Mother/infant	+	+	+	+	+	+
**Brutus**/Ali	-	+	-	+	+	+
**Brutus**/Tarzan	-	+	-	+	+	+
**Ulysse**/Brando	-	+	-	-	+/−	+
**Fredy**/Yayo	?	?	+	+	+	-
**Fredy**/Carim	+	?	+	+	+	-
**Porthos**/Gia	-	+	+	+	+	+
**Fredy**/Victor	+	+	+	+	+	+

**Figure 3:**
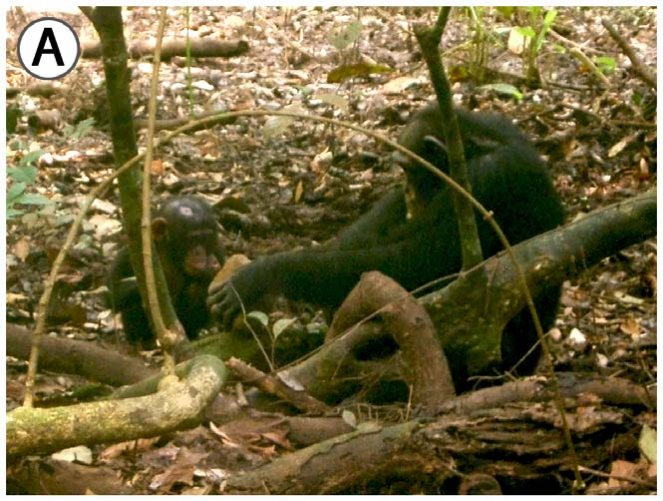
The adult male Porthos with his adopted female infant Gia. A) Porthos cracking and sharing nuts with Gia, B) Porthos carrying Gia on his back.

**Figure 4:**
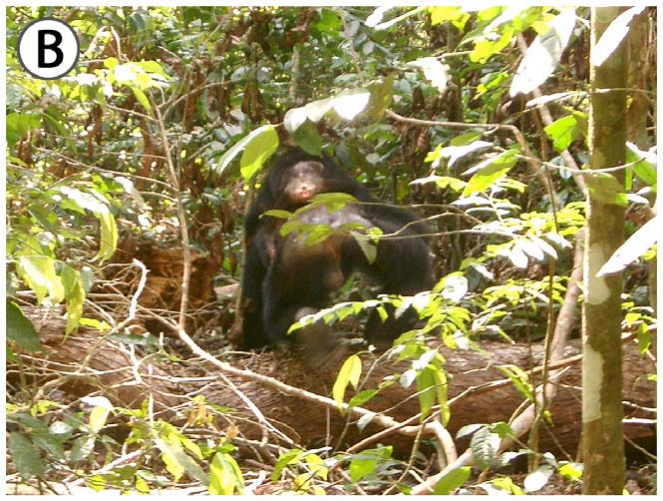
The adult male Porthos carrying on his back the adopted female infant Gia.

Remarkably, all adult males of the East Group that adopted young orphans went a step further by investing in unweaned small infants and carrying them dorsally during travel for many months (see Figures 3 and 4 of Porthos with Gia) (Table 3). Since, Taï chimpanzees walk about 8 km per day on average, this represents a notable investment. Porthos' adoption of Gia lasted for 17 months, until his death due to Anthrax, and he was seen to carry her even in extremely risky situations, such as during encounters with neighboring communities [26]. Furthermore, some males were seen to share their night nest with their adopted infant (Table 3). Fredy, the 3^rd^ ranking male of the East Group, adopted Victor, the son of Vanessa, who died from Anthrax in late December 2008, and shared his nest with him every night, carried him on his back for all long travels, and shared the Coula nuts he opened from December 2008 to July 2009. For example, on February 17th, Fredy cracked 196 Coula nuts for 2h05mn and shared pieces of 79% of them. This gives a measure of the altruistic investment made in an unrelated infant.

These adoptions by adult males of orphans that are often not their own offspring plainly show that, contrary to earlier sweeping conclusions [9]–[10], [12], chimpanzees are sensitive to the welfare of unrelated group members. Adoption, which is not uncommon in the animal kingdom, including in humans, is normally explained by close genetic relatedness [27]–[29], so the adoption of unrelated orphans by adult males is notable. Nevertheless, might chimpanzee males gain some indirect benefit from investing so heavily in unrelated infants? One potential long-term benefit of adoption by adult males is that once an orphan becomes an adult, 10 years later, he could become an ally of the aging male. This might have happened in the case of Ali, had he survived longer. However, this certainly does not apply to adoption of female orphans. Economists have proposed that, in humans, either the improved reputation of the altruistic individual might compensate for the costs or that a social normative rule, in the form of punishment by one's peers, imposes such behaviors [3], [33]. Punishment seems unlikely in the case of adoption of small orphans and we have not seen this to occur. On the other hand, improved reputation might play a role, but we have not yet determined that males who adopt orphans are more reproductively successful, as should have been the case for Brutus, the male that adopted two orphans, and Ulysse [32], [34].

The observations of adoptions in Taï chimpanzees support our proposition, emerging from Hamilton's rule, that altruism is not hard wired and will be directed specifically towards individuals that profit significantly from this act. The fact that we could not show that adopted orphans have higher survival rates than non-adopted orphans (Figure 2) is most likely due to the high mortality rates in the Taï chimpanzee study groups for over 2 decades, which may easily overshadow potential beneficial effects such as this [35]–[36]. By guaranteeing that all individuals have a safe environment and access to food, captive situations might not mimic situations in which the welfare of others is an issue. Altruism in the case of adoption in forest chimpanzees seems to be the outcome of the specific socio-ecological conditions faced by the individuals. The high level of adoption observed in Taï chimpanzees compared to other well-studied East African populations might result from the fact that the Taï population coexists with a large population of leopards and the resulting high predation pressure exerted by these cats seems to have promoted strong within-group solidarity in the form of care for all injured individuals as well as joint coalition defense against the leopards [16], [26]. Once established, this care for the welfare of others seems to have been generalized to new social contexts, including adoption [26]. Any discussions about the evolution of altruism must include the caveat that dissimilar socio-ecological conditions will lead to important population differences in both chimpanzees and humans and we need to remain very careful before making any claims about species differences.

## Methods

### Ethics Statement

This research complied with the ethics guidelines of the Max Planck Society and was supported by the Ivorian authorities (Office Ivoirien des Parcs et Réserves and the Ministry of Science and Research of Côte d'Ivoire).

### Details of the Data Collection Procedures

Observations of chimpanzees in the Taï National Park, Côte d'Ivoire, have been done for three decades with up to four neighbouring communities [16]–[17], [26], [29]. Observations on the fully habituated North and South communities have been ongoing since 1982 and 1993, respectively. The Middle group was fully habituated in 1995 and remained under constant observation until summer 2004, at which time we reduced observations to one week every three months to update our demographic records because the community had been reduced to five individuals. No orphan was observed in this group during the 9 years we followed them continuously. In 2000, the habituation of the East group was initiated and by February 2005, at least 11 adult males and 12 adult females were known to be present.

Demographic information on all groups was collected on a daily basis by a team of trained field assistants and students. In addition, detailed data collection was done using standardized check sheets recording party composition, party size, any changes in party composition, as well as basic social interactions. Detailed data on adoptions were collected *ad libitum* as this event took place, as well as on our mother-infant check-sheets. The further development of the adoption process was complemented by information recorded on the social interactions check sheet when the adopter was a target and this included data on food sharing, support in aggression, and co-nesting.
